# Aggregate Labor of Mankind

**Published:** 1864-12

**Authors:** 


Aggregate Labor of Mankind.—Along with the compassion that is excited by listening to a tale of want, there is apt to arise, at the same time, a feeling of astonishment that such a thing should *be* in a land like this. Perhaps, however, the true wonder is that want is not universal. One-half of the race die before they have contributed an iota to the world's sustenance or their own. One-half of those who survive the period of childhood are women, who do not, as a general thing, contribute directly to the production of wealth. Of the men, many are sick, many are old, many are idle, many are wasteful, many are parasites. Those who do work and live to the age of three score years and ten, spend one-third of their lives in bed, one-twentieth at the table, one-sixth in recreation. Much of their time is wasted in mistakes; much of what they succeed in producing is swept away by fire and flood. During half of the year Nature sleeps. One harvest in five proves a failure. Only a fraction of the earth's surface is capable of cultivation. A large part of the general labor is absorbed in the production of luxuries, in repairing the damages of war, in preparing for future conflicts, in the transportation of produce, and in journeys. Probably not more than *one-tenth* of the whole amount of human force is expended in earning the world's daily bread. The standing marvel, therefore, of society is, not that any should suffer want, but that there should be any who do not.—*Home Journal.*

